# Proposed Technique for Accurate Detection/Segmentation of Lung Nodules using Spline Wavelet Techniques

**Published:** 2013-03

**Authors:** T. K. Senthil Kumar, E. N. Ganesh

**Affiliations:** 1 *Department of ICE Anna University Of Technology, Chennai, India;*; 2 *Rajalakshmi Institute of Technology, Anna University, Chennai, India*

**Keywords:** lung nodules, spline, wavelets, medical image segmentation

## Abstract

In this paper we are going to discuss and analyze the different methods which are developed to detect the Lung nodules which cause the lung cancer. At the end of analyzing different methods, the new methodology of detecting the lung nodules using Spline Wavelet technique has been proposed in this paper. Continuous modeling of data often required in medical imaging, Polynomial Splines are especially useful to consider image data as continuum rather than discrete array of pixels. The multi resolution property of Splines makes them prime candidates for constructing wavelet bases. Wavelet tool also let us to compress the original CT image to greater factor without any sacrifice in accuracy of nodule detection. Different Algorithms for segmentation/ detection of lung nodules from CT image is discussed in this paper.

## INTRODUCTION

Lung cancer is one of the serious cancer which causing more death world wide. Statistics says 28 percent of overall cancer deaths are due to lung cancer. It is the number one cause of death from cancer every year and the second most diagnosed after breast and prostate cancers (for women and men, respectively). Lung cancer is usually found in older persons because it develops over a long period of time. Lung cancer is usually visible as small round lesion called ‘nodules’ through Medical images like CT. However the major problem in identifying the nodules with CT image is that the characteristics of nodules are similar to the characteristics of blood vessels and bronchi in terms of size, shape and density. Hence it is necessary to enhance and segment the nodules using some special Image processing techniques. Early detection of lung cancer using the CT image has the largest chance of saving the patient’s life. It is important to enhance and detect the nodules in CT images in order to identify the lung cancer at early stage. Different methods and algorithms are developed to effectively detect the nodules.

In this paper the various methods are discussed and one new method is proposed for efficient nodules detection.

## PREVIOUS RELATED WORKS

Initially some works had been carried out to detecting the nodules with the help of X-ray (1). X-ray may show a mass in the lungs or enlarged lymph nodes. Sometimes the chest x-ray is normal, and further tests are needed look for a suspected lung cancer. Even if a mass is found, these are not always cancerous and further studies are needed. Hence researchers start to work with CT scan image which have more information about the tissues of body part compare to X-ray Some other medical images like MRI and PET may have much more useful information than CT, but because of the cost factor CT lead the diagnosis process. Hence whatever advancement and enhancement carried on CT images will help the people greatly. In this section we study some of the research carried out with the CT images of lungs to find the lung nodules.

### 3-D Vascular method of Lung Nodule Classification

This method includes the enhancement of lung structures followed by series of segmentation methods to extract the nodule and to form a 3D configuration at an area of interest (nodule) (2). The vascular index, aspect ratio, circularity, irregularity, extent, compactness and convexity were also computed as shape features for quantifying the nodule boundary. The main disadvantage of the 3D methods are it takes more computing time.

### Enhancement and Detection of lung modules with Multi-scale filters

**Figure 1:**
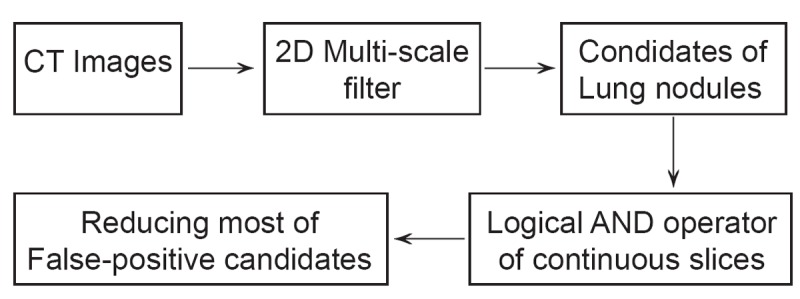
Two step method for lung nodules detection in CT images

In this method Takemura, Han, chen, Ito and Nishikwa (3) proposed a two step method for lung nodules detection in CT images. Firstly they used 2D multi-scale filter to detect the candidates of lung nodules on the slice images and then reduce the most of false positive nodules using logical AND operator of continuous slices (Fig. 1).

**Figure 2:**
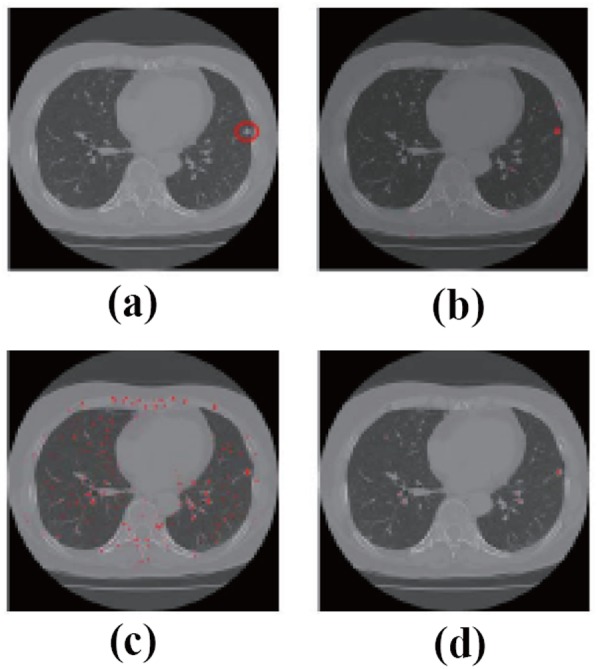
a, Original CT Image; b, Enhanced nodules by 3D multi-scale filter; c, 2D Multi-scale filter; d, Takemura’s proposed method

They take a slice of an original CT image, which has a lung nodule, as shown in Fig. 2a, therein, red circle shows the location of the true-positive nodule. In Fig. 2a, the enhanced nodules by a 3D multi-scale filter, a 2D multi-scale filter and the proposed method are shown in Fig. 2b-2d, respectively.

**Figure 3:**
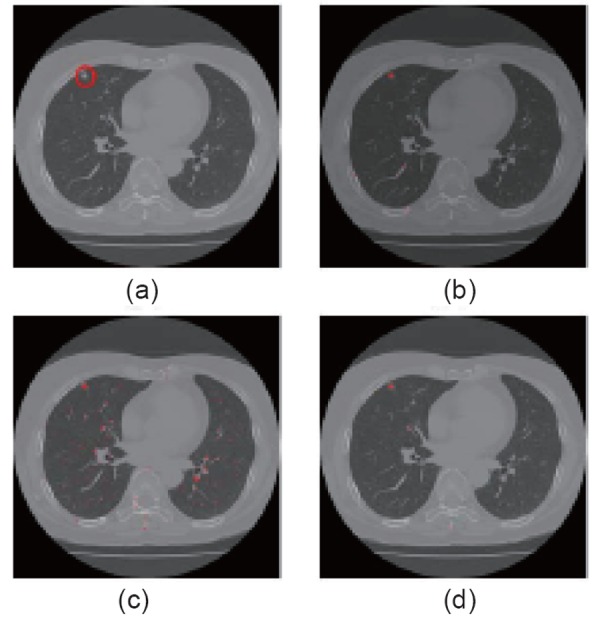
a, Original CT Image; b, Enhanced nodules by 3D multi-scale filter; c, 2D Multi-scale filter; d, Takemura’s proposed method

In Fig. 3, they showed another example. From Fig. 2 and Fig. 3, it is obvious that the results of 2D multi-scale filter retain too much false positive nodules. The results of 3D multi-scale filter and this proposed approach got comparable good results. However, 3D multi-scale filter takes so much time and it is not applicable in real system, while this method can only need the time similar to 2D multi-scale filter method. In case of 31 slices, window size is 21 and scale 4, 3D multi-scale filter takes 1.2 day to process, while this proposed method takes only 15 min.

The objective of this method proposed by Anitha and Sridhar (4) is to develop a segmentation system in order to assist the surgeons to remove the portion of lung for the treatment of certain illness such as lung cancer, and tumors.

**Figure 4:**
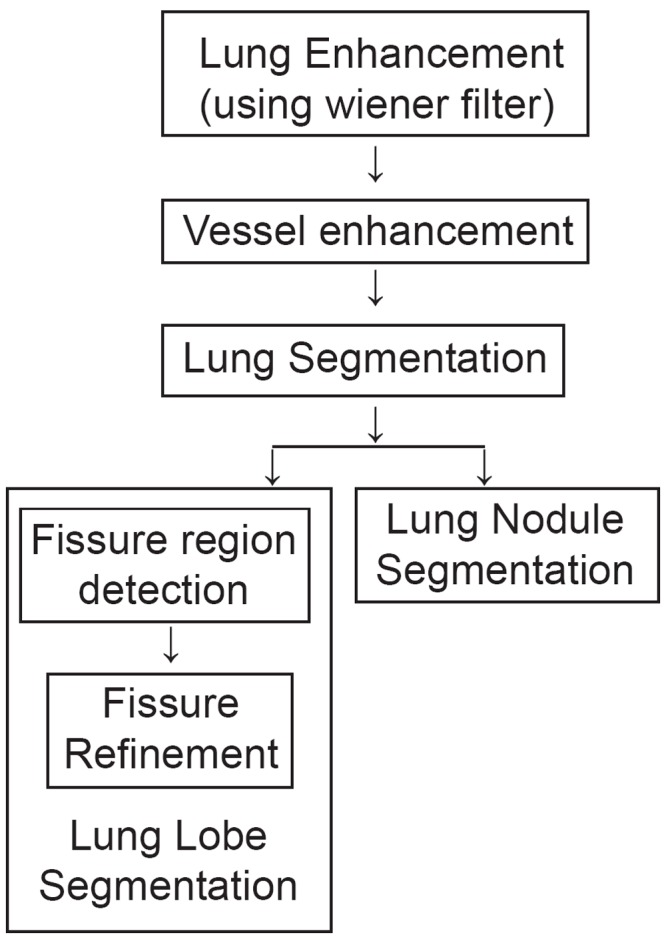
system architecture

This method having following steps:

- Image Enhancement and Segmentation.
- Fissure Detection for identifying the fissure regions.
- Fissure Refinement using region growing method.
- Nodule Segmentation using Adaptive Threshold

The system architecture is presented in Figure 4.

In this method initially pre-processing of a CT image is done to remove the noises present in it. Then the vessels are enhanced using the morphological operators followed by lung segmentation. The fissure regions are identified, enhanced and verified. Lung nodules are segmented using adaptive threshold. Through the project they have developed a segmentation algorithm for identifying the fissures and nodules from CT images. Using the statistical analysis such as mean difference between the automatic and manual segmentation, the results are verified. These statistical results are tabulated in Table 1.

**Table 1:** Results for identifying the fissures and nodules from CT images

Patient #	Manual Segmentation M_a_	Automatic Segmentation A_a_	Mean Difference M_d_=abs(M_a_-A_a_)	Accuracy % (100*M_d_)/(M_a_+A_a_)

1	220	161	59	87
2	191	161	30	92
3	1654	3661	2007	72.58
4	1408	4263	2855	66.5
5	639	3274	2635	60
6	161	214	53	88
7	84	85	31	82
8	1270	4133	2863	65.3

## SPLINE WAVELETS

Researchers are now faced with an ever increasing variety of wavelet bases to choose from. While the choice of the "best" wavelets obviously application-depend, it can be useful to isolate a number of properties and features that are of general interest to the user. The purpose of this section is to present a list of arguments in favor of splines, which are unique in a number of ways.

Wavelets can be classified in four categories: orthogonal (Battle-Lemarié), semi-orthogonal (e.g., B-spline), shift-orthogonal, and biorthogonal (Cohen-Daubechies-Feauveau). Unlike most other wavelet bases, splines have explicit formulae in both the time and frequency domain, which greatly facilitates their manipulation. They allow for a progressive transition between the two extreme cases of a multiresolution (5): Haar’s piecewise constant representation (spline of degree zero) versus Shannon’s bandlimited model (which corresponds to a spline of infinite order). Spline wavelets are extremely regular and usually symmetric or anti-symmetric. They can be designed to have compact support and to achieve optimal time-frequency localization (B-spline wavelets). The underlying scaling functions are the B-splines, which are the shortest and most regular scaling functions of order *L*. Finally, splines have the best approximation properties among all known wavelets of a given order *L*. In other words, they are the best for approximating smooth functions.

### Spline wavelet in Medical Image processing

Polynomial splines are especially useful when one wishes to consider image data as a continuum rather than a discrete array of pixels. Such a continuous modeling of the data is often required in medical imaging. Interpolation, in particular, plays a crucial role at various stages of processing. For instance, it is present-explicitly or not-for tomographic reconstruction, irrespective of the type of algorithm used (filtered back-projection, inverse Fourier or iterative reconstruction). Another important area is medical image visualization; this involves simple 2D operations such as image zooming, panning, rotation, or 3D manipulations, such as reslicing or maximum intensity projection 29, which are often used by radiologists. Interpolation models are also required for performing various types of image registrations 10, 47; these include intra-modal registration for rigid-body motion compensation, inter-modal registration of CT, PET and MR data sets of a same subject. Considering an image as a continuously–defined function is also often desirable for feature extraction, in particular, contour detection. These are all examples of medical imaging tasks that can benefit from the use of splines; a more complete inventory is given in Table 2.

**Table 2:** Summary of medical image application using spline wavelets

Image processing task	Specific operation	Imaging modality

Tomographic reconstruction	·Filtered backprojection	Commercial CT (X-rays)
	·Fourier reconstruction	EM
	·Iterative techniques	PET, SPECT
	·3D + time	Dynamic CT, SPECT, PET
Sampling grid convertion	·Polar-to-cartesian coordinates	Ultrasound (endovascular)
	·Spiral sampling	Spiral CT, MRI
	·k-space sampling	MRI
	·Scan conversion	
Visualization	2D operations	
	·Zooming, parnning, rotation	All
	·Re-sizing, scaling	
	·Stereo imaging	Fundus camera
	·Range, topography	OCT
	3D operations	
	·Re-slicing	CT, MRI, MRA
	·Max. intensity projection	
	·Simulated X-ray projection	
	Surface/volume rendering	
	·Iso-surface ray tracing	CT
	·Gradient-based shading	MRI
	·Stereogram	
Geometrical correction	·Wide-angle lenses	Endoscopy
	·Projective mapping	C-Arm fluoroscopy
	·Aspect ratio, tilt	Dental X-rays
	·Magnetic field distortions	MRI
Registration	·Motion compensation	fMRI, Fundus camera
	·Image subtraction	DSA
	·Mosaicking	Endoscopy, fundus camera
	·Correlation-averaging	EM microscopy
	·Patient positioning	Surgery, radiotherapy
	·Retrospective comparisons	
	·Multi-modality imaging	CT/PET/MRI
	·Stereotactic normalization	
	·Brain warping	
	·Contours	All
	·Ridges	
	·Differential geometry	
	*Contour extraction*	
	·Snakes and active contours	MRI, Microscopy (cytology)

Our purpose is to justify the use of splines in imaging applications, emphasizing their ease of use, as well as their fundamental properties. Modeling images with splines is painless: it essentially amounts to replacing the pixels by B-spline basis functions, which are piecewise polynomials with a maximum order of differentiability.

The spline representation is flexible and provides the best cost/quality tradeoff among all interpolation methods: by increasing the degree, one shifts from a simple piecewise linear representation to a higher order one that gets closer and closer to being bandlimited. Multiresolution properties of splines make them especially attractive for multi-scale processing. On the more fundamental front, we will show that splines are intimately linked to differentials; in fact, the B-splines are the exact mathematical translators between the discrete and continuous versions of the operator. This is probably the reason why these functions play such a fundamental role in wavelet theory. Splines may also be justified on variational or statistical grounds; in particular, they can be shown to be optimal for the representation of fractal-like signals.

### Mathematical model of Polynomial spline

A polynomial spline (6) of degree *n* is made up of polynomial segments of degree *n* that are connected in a way that garantees the continuity of the function and of its derivative up to order *n*-1. The joining points between the polynomial segments are called *knots*. In the context of the wavelet transform, the knots are equally-spaced and typically positioned at the integers. One can thus define a hierarchy of spline subspaces of degree *n,* {*Vi* ^n^} *i*∈*Z*,

where *Vi^n^* is the subspace of *L*2-functions that are (*n*-1) times continuously differentiable and are polynomials of degree *n *in each interval (2^i^ *k*, 2^i^(*k*+1)), *k*∈*Z*. The spacing between the knotpoints 2^i^ is controlled by the scale index *i*. Clearly, a function *f(x)*∈*Vi0* ^n^ that is piecewise polynomial on each segment(2^io^ *k*, 2^io^(*k*+1)) is also included in any of the finer subspaces* Vi* ^n^ with *i*∈*i*0. Thus, we have the following inclusion property

$$\{0\}\subset \ldots V_{1}^{n}\subset \ldots V_{0}^{n}\subset V_{-1}^{n}\subset \ldots \subset L_{2}$$

Furthermore, it is well known that one can approximate any *L*2-function by a spline as closely as one wishes by letting the knot spacing (or scale) go to zero ( *i*→ - infinity). This means that the above sequence of nested subspaces is dense in *L*2 and therefore meets all the requirements for a multiresolution analysis of *L*2 in the sense defined by Mallat. This implies that it is indeed possible to construct wavelet bases that are polynomial splines. The best way to proceed is to use Schoenberg's representation of splines in terms of the B-spline basis functions. In order to satisfy the multiresolution inclusion property for any degree *n*, we will use the so-called *causal B splines* which can be constructed from the (*n*+1)-fold convolution of the indicator function in the unit interval (causal B-spline of degree 0)

$$\varphi^{n}\left(x\right)=\begin{array}{c} \varphi^{0}*...*\varphi^{0}\left(x\right)\\ \overset{︷}{\left(n+1\right)\;times} \end{array}$$

where

$$\varphi^{0}\left(x\right)=\left\{ \begin{array}{c} 1,\;0\leq x\leq 1\\ 0,\;otherwise \end{array}\right.$$

The B-spline of degree n satisfies the two-scale relation

$$\varphi^{n}\left(x/2\right)=\sqrt{2}\underset{k\in z}{\Sigma }h^{n}\left(k\right)\varphi^{n}\left(x-k\right)$$

where h^n^(*k*) is the binomial filter of order *n*+1 whose transfer function is

$$h^{n}\left(k\right)\overset{z}{↔}H^{n}\left(z\right)=\sqrt{2}{\left(\frac{1+z^{-1}}{2}\right)}^{n+1}$$

In 1946, Schoenberg proved that any polynomial spline of degree *n* with knots at the integers could be represented as a linear combination of shifted B-splines. Thus, our basic spline space *Vo* ^n^ can also be specified as

$$V_{0}^{n}=\left\{s_{0}\left(x\right)=\underset{k\in z}{\Sigma }c\left(k\right)\varphi^{n}\left(x-k\right)|c\in l_{2}\right\}$$

where the weights *c*(*k*) are the so-called B-spline coefficients of the spline function *s*_0_(x). In addition, it can be shown that the Bsplines {φ^n^ (x-k)}_k∈z_ constitute a Riesz basis of *Vo* ^n^ in the sense that there exist two constants *An* >0 and *Bn* < + infinity such that

$$\forall c\in l_{2},\;A_{n}\cdot {||c||}_{l_{3}}^{2}\leq {||\underset{k\in z}{\Sigma }c\left(k\right)\varphi^{n}\left(x-k\right)||}_{l_{2}}^{2}\leq B_{n}\cdot {||c||}_{l_{2}}^{2}$$

The lower inequality implies that the B-splines are linearly independent (i.e., *s*_0_(x)=0 → *c*(*k*)=0). The upper inequality guarantees that *Vo* ^n^∈L_2_. Hence, any polynomial spline has a unique representation in terms of its B-spline coefficients *c* (*k*).Schoenberg also proved that the B-splines are the shortest possible spline functions. This, together with the fact that thesefunctions have a simple analytical form, makes the B-spline representation one of the preferred tools for the study andcharacterization of splines.

### Mathematical model of Bi-orthogonal Wavelets

In the most general case, the construction of biorthogonal wavelet bases involves two multiresolution analyses of *L*2: one for the analysis, and one for the synthesis 7. These are usually denoted by

*Vi i*{(φ˜)}_icz_   and *Vi i*{(φ)}_icz_

where φ˜(*x*) and φ(*x*) are the analysis and synthesis scaling functions, respectively. Note that φ˜(*x*) and φ(*x*) can be arbitrary solutions of a two-scale relation and not necessarily the causal B-splines φ*n* defined previously. The corresponding analysis and synthesis wavelets ψ˜ (*x*) and ψ(*x*) are then constructed by taking linear combinations of these scaling functions.

$$\overset{˜}{\Psi }\left(x/2\right)=\sqrt{2}\sum_{k}\overset{˜}{g}\left(k\right)\overset{˜}{\varphi }\left(x-k\right)$$

$$\Psi \left(x/2\right)=\sqrt{2}\sum_{k}g\left(k\right)\varphi \left(x-k\right)$$

They form a biorthogonal set in the sense that

$$<\Psi_{i,k},\;{\overset{˜}{\Psi }}_{f,i}>=\delta_{i-f,k-i}$$

with the short form convention ψ_*I,k*_= 2^i/2^ ψ (2^i^ x-k). T his allows us to obtain the wavelet expansion of any *L*2-function as

$$\forall f\in L_{2},\;f=\underset{l\in z}{\Sigma }\;\underset{k\in z}{\Sigma }\left(f,{\overset{˜}{\Psi }}_{l,k}\right)$$

Note that the underlying basis functions are usually specified indirectly in terms of the four sequences *h*(*k*), *h*˜(*k*), *g*(*k*) and *g*˜(*k*), which are the filters for the fast wavelet transform algorithm.

### Mathematical model of Spline Wavelets

We have a spline wavelet transform whenever the synthesis functions (ψ(*x*) and φ(*x*)) are polynomial splines of degree *n*. This means that the synthesis wavelet can also be represented by its B-spline expansion

$$\Psi \left(x/2\right)=\underset{k\in z}{\Sigma }w\left(k\right)\varphi^{n}\left(x-k\right)$$

It is important to observe that the underlying scaling function φ(*x*)∈*Vo* ^n^ is not necessarily the B-spline of degree *n* — unless *h*(*k*) is precisely the binomial filter. This function is usually specified indirectly as the solution of the two-scale relation

$$\varphi \left(x/2\right)=\sqrt{2}\underset{k\in z}{\Sigma }h\left(k\right)\varphi \left(x-k\right)$$

where *h*(*k)* is the corresponding (lowpass) reconstruction filter. However, in the spline case, there will always exist a sequence *p*(*k*) such that

$$\varphi \left(x\right)=\underset{k\in z}{\Sigma }p\left(k\right)\varphi^{n}\left(x-k\right)$$

Such specific B-spline characterizations for various kinds of spline scaling functions (orthogonal, dual, or interpolating) can be found elsewhere. Note that the sequence *p*(*k*) defines an invertible convolution operator from *l*2 into *l*2 which performs the change from one coordinate system to the other (i.e., φ to φ*n*). The basic requirement for φ(x-k) to form a Riesz basis of *Vn*0 is that there exist two constants *Ap* > 0 and *Bp* < +∞ such that *Ap < | P(e ^jw^)^2^| <B p almost everywhere*, where *P(e^jw^) * denotesthe Fourier transform of *p*.

By combining above φ(*x*) equation with Bi-orthogonal ψ(*x*/2) equation, we obtain the B-spline coefficients of the wavelet ψ(*x*):

$$w\left(k\right)=(p*g)\left(k\right)\overset{z}{↔}W\left(z\right)=P\left(z\right)G\left(z\right)$$

**Figure 5:**
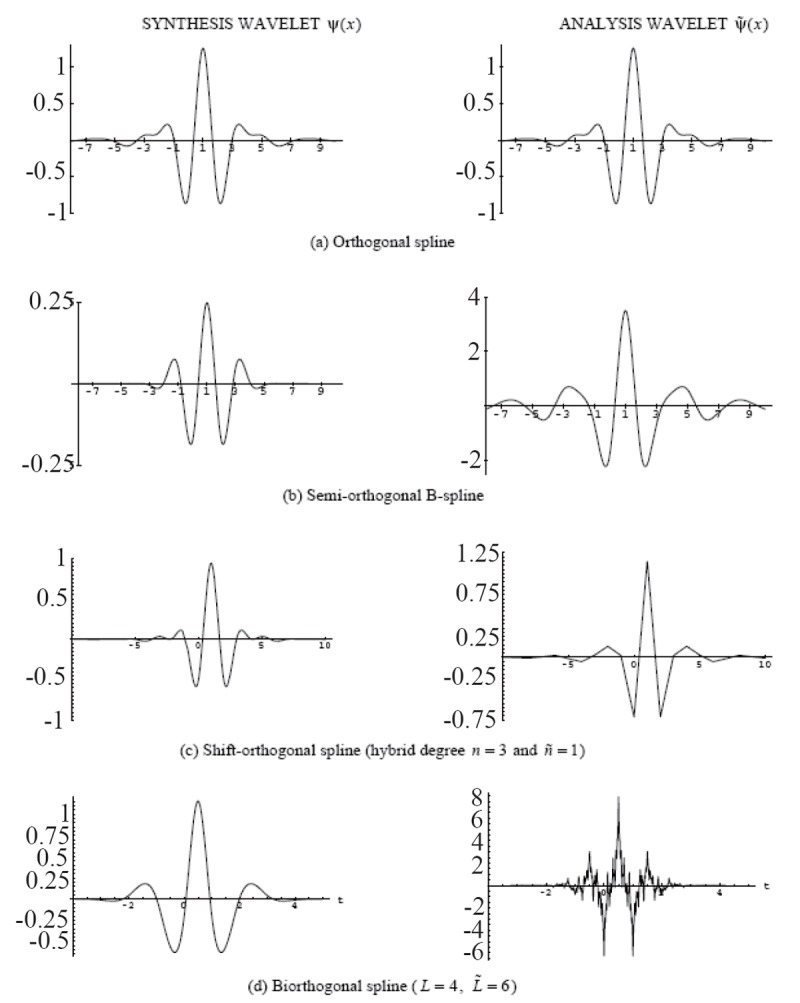
Examples of four different types of cubic splines wavelets and their corresponding duals

Many kinds of spline wavelets have been described in the literature. The four primary types can be differentiated on the basis of their orthogonality properties; they are summarized in Table 3. Four corresponding examples of cubic spline wavelets and their duals are also shown in Figure 5.

**Table 3:** Classification of Spline wavelets with its main properties

Wavelet type	Orthogonality	Compact support	Key properties	Implementation

Orthogonal splines (Battle-Lemarie, Mallat)	Yes	No	* Symmetry & regularity	IIR/IIR
			+ Orthogonality	
Semi-orthogonal splines (B-splines) (Chui-Wang, Unser-Aldroubi)	Inter-scale	Analysis or Synthesis	* Symmetry & regularity	Recursive IIR/FIR
			+ Optimal time-frequency localization	
Shift-orthogonal splines (Unser-Thevenzaz-Aldroubi)	Intra-Scale	No	* Symmetry & regularity	IIR/IIR
			+ Quasi-orthogonality	
			+ Fast decaying wavelet	
Biorthogonal splines (Cohen-Daubechies-Feauveau)	No	Yes	* Symmetry & regularity	FIR/FIR
			+ Compact support	

## PROPOSED METHOD

The method that we proposing in this paper consist of three following major steps

1. De-noise the CT Image using un-decimated Wavelet transform;
2. Compress the CT image using Spline Bi-orthogonal Wavelet;
3. Accurate detection of nodules with the compressed image of step 2 using spline wavelets.

### De-noise using un-decimated Wavelet transform

Developing Image denoising algorithms is a difficult task since fine details in a medical image embedding diagnostic information should not be destroyed during noise removal. Many of the wavelet based denoising algorithms use DWT (Discrete Wavelet Transform) in the decomposition stage which is suffering from shift variance. To overcome this in this paper we are proposing the denoising method which uses Undecimated Wavelet Transform to decompose the image and we performed the shrinkage operation to eliminate the noise from the noisy image. In the shrinkage step we used semi-soft and stein thresholding operators along with traditional hard and soft thresholding operators and verified the suitability of different wavelet families for the denoising of medical images.

Naga Prudhvi Raj and venkateswaralu (7) applied this un-decimated wavelet for denoising the leg knee CT images and their statistical results are tabulated below in Table 3. Table has the Mean square error (MSE) and Peak Signal to Noise Ration (PSNR) values for different sigma values.

Hard, soft, semi-soft and stein are different Thresholding criteria.

Based on the analysis of the above result we proposed to use this Undecimated wavelet approach for de-noising the lung CT image before applying the nodule detecting algorithm.

### Compression using Spline Bi-orthogonal Wavelet

With extensive digitization of data and increasing use of Computer Aided Diagnosis, most of image data in hospitals are stored in digital form using picture archiving and communication systems. The need for data storage and bandwidth requirements is increasing and lossy compression techniques have become a necessity. The successful use of the wavelet transform in the field of image compression has been extensively studied in literature. We proposed to use the Biorthogonal spline wavelet (8) as an effective mode for medical image compression. The research made by Jane P and his team at Thoracic division, Department of Radiology, New York University (9) medical center achieved the 1:10 ratio of compression without any sacrifice in the nodule detection.

Their results are tabulated as follow:

The above results make sure that Bi-orthogonal wavelet method is suitable for compressing the lung images before applying the nodules segmentation technique. In the above research they used some filtering methods for nodules detection, but in this proposed method we are going to use the Spline techniques to detect the nodules in the lungs. As we discussed in section 3, the spline wavelet techniques are optimum for medical image segmentation and object detection. The globe round shaped nodules can be efficiently detected using B-spline wavelet. The first step of our proposed method Denoising lead to accurate detection of lung nodules at step 3 and also the compression of images that we proposed in step 2, reduce the computing time of step 3. Hence in terms of accuracy and time this proposed method is optimum.

## CONCLUSION

Many Nodule detection algorithms were surveyed and discussed in this paper. After analyzing the different methods, we proposed the optimum method for detecting the nodules in lungs which comprise of 3 steps say denoising, compression and Spline based nodule detection respectively. It has been proposed to use Un-decimated wavelet for Denoising, Bi-orthogonal wavelet method for Compression and B-Spline Wavelet technique for Nodule detection after analyzing many previous research and literatures.

## References

[1] Jun WEI, Yoshihiro Hagihara, Akinobu Shimizu, Hidefumi Kobatake Optimal image feature set for detecting lung nodules on chest X-ray images. CARS 2002 – H.U. Lemke Springer.

[2] Classification of Lung nodules in diagnostic CT: an approach on 3D vascular. NHI/NCI Grants Sponsor.

[3] Takemura Han, Chen K, Ito Nishikwa, Ito M (2008). Enhancement and detection of lung nodules with Multiscale filters in CT images. International Conference on Intelligent Information, IEEE.

[4] Anitha S, Sridhar S (2010). Segmentation of Lung Lobes and Nodules in CT Images. SIPIJ.

[5] Michel Unser (2002). Splines: a perfect fit for medical imaging. SPIE symposium on Medical Imaging.

[6] Michel Unser (1997). Ten Good Reasons for using Spline Wavelets. Wavelets Applications in Signal and Image Processing V. Proc. SPIE.

[7] Naga Prudhvi Raj, venkateswaralu (2011). Denoising of Medical Images Using Undecimated Wavelet Transform.

[8] Loganathan R, Kumaraswamy YS (2010). Medical Image Compression Using Biorthogonal Spline Wavelet with Different Decomposition. IJCSE.

[9] Jane P, Henry David Wavelet Compression of Low-Dose Chest CT Data: Effect on Lung Nodule Detection.

